# Glioblastoma Multiforme in the Posterior Cranial Fossa in a Patient with Neurofibromatosis Type I

**DOI:** 10.1155/2009/757898

**Published:** 2009-12-16

**Authors:** Marike L. D. Broekman, Roelof Risselada, JooYeon Engelen-Lee, Wim G. M. Spliet, Bon H. Verweij

**Affiliations:** ^1^Rudolf Magnus Institute of Neuroscience, University Medical Center Utrecht, 3508 GA Utrecht, The Netherlands; ^2^Department of Neurosurgery, University Medical Center Utrecht, 3508 GA Utrecht, The Netherlands; ^3^Department of Medical Informatics, Erasmus University Medical Center, 3051 GE Rotterdam, The Netherlands; ^4^Department of Pathology, University Medical Center Utrecht, 3508 GA Utrecht, The Netherlands

## Abstract

Patients with Neurofibromatosis type 1 (NF1) have an increased risk of developing neoplasms. The most common brain tumors, found in 15%–20% of NF1 patients, are hypothalamic-optic gliomas, followed by brainstem and cerebellar pilocytic astrocytomas. These tumors generally have a benign nature. NF1 patients are predisposed to a 5-fold increased incidence of high-grade astrocytomas, which are usually located in supratentorial regions of the brain. We present an NF1 patient who developed a high-grade astrocytoma in the posterior fossa and discuss possible pathophysiological mechanisms.

## 1. Introduction

Neurofibromatosis type 1 is the most common neurocutaneous disease with a prevalence of approximately 1 in 2600 to 1 in 3000 individuals [1]. The majority of Neurofibromatosis type 1 (NF1) patients are only mildly affected, but the clinical course is highly variable, though generally progressive [2]. The cardinal feature of NF1 is the development of multiple neurofibromas, with a 10% lifetime risk of progressing to malignant peripheral nerve sheath tumors (MPNSTs) [3]. Other characteristics include (in order of appearance) café au lait spots, skin fold freckles, Lisch nodules, and skeletal anomalies [4]. In addition, NF1 patients have an increased risk of developing neoplasms, such as leukemia, pheochromocytomas, and brain tumors [5, 6]. The most common of these are hypothalamic-optic gliomas, followed by brainstem and cerebellar pilocytic astrocytomas (see for a review [7]). 

The astrocytomas observed in 15%–20% of these patients generally have a benign nature [8]. NF1 patients however have a 5-fold increased incidence of high-grade astrocytoma (WHO grade IV), also known as glioblastoma multiforme (GBM) [9]. 

These tumors are usually located in supratentorial regions of the brain. 

We present a 28-year-old female NF1 patient who developed a GBM in the posterior cranial fossa and discuss the possible pathophysiological mechanisms. 

## 2. Case Report

### 2.1. History and Presentation

The patient, a 28-year-old woman with NF1 was regularly seen for follow-up of a cerebellar lesion, suspected to be a hamartoma. This lesion was accidentally discovered during a radiological exam performed for a facial neurofibroma, when she was 21 years old. Successive MRI-scans of the brain were performed. 

At the age of 28, she was referred to our hospital because of complaints of progressive balance disturbance, ataxia of the right arm and leg, diplopia, and a sense of pulsation at the back of her head. She mentioned that a “bump” at the back of her head had increased in size over the preceding months.

Neurological examination revealed nystagmus of her right eye, diplopia, loss of sensibility of the face, and ataxia of the right arm and leg. Romberg's test was positive.An occipital/cerebellar solid mass of 4 by 5 cm was palpable. 

**Figure 1:**
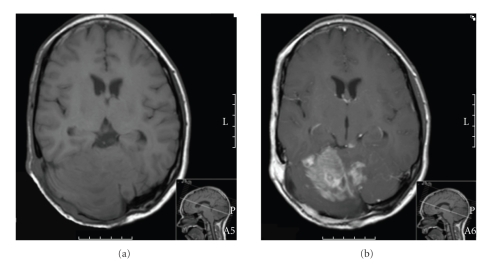
T1-weighted axial MRI-scan of the brain. T1-weighted axial MRI showed a (sub)occipital right-sided bone defect and a lesion in the right cerebellar hemisphere (a) that enhanced after i.v. Gadolinium administration (b).

An MRI-scan of the brain (Figures 1(a) and 1(b)) showed a (sub)occipital right-sided bone defect, contiguous to a subcutaneous neurofibroma, and a lesion in the right cerebellar hemisphere that enhanced after Gadolinium administration (Figure 1(b)). In the left cerebellar hemisphere and the left superior colliculus other lesions were found, that both also enhanced upon Gadolinium administration.

### 2.2. Treatment, Histological Evaluation and Postoperative Course

**Figure 2:**
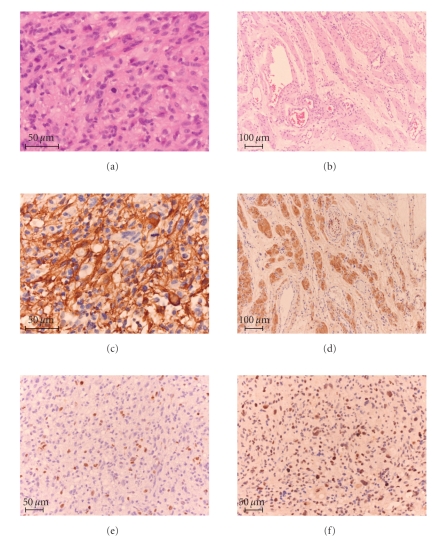
Histopathological and immunohistochemical findings. Histology of the first lesion showed a cellular astrocytic neoplasm (H&E) (a). The second lesion impressed as a neurofibroma (H&E) (b). The first lesion was GFAP positive (c), whereas the second was not (data not shown). This lesion was however positive for S100 (d). ~10%–15% of the nuclei of the first lesion were immunohistochemically stained for Ki-67 (MiB1) (e). In this lesion, approximately 30% of the cells showed immonupositivity for p53 (f).

Neurosurgical resection of the lesion was subsequently performed. Histological analysis revealed two distinct lesions: a mitotically active pleomorphic astrocytoma with pathologic vascular proliferation that was classified as glioblastoma multiforme (astrocytoma, grade 4) (Figures 2(a), 2(c), 2(e), and 2(f)) and a diffuse neurofibroma (Figures 2(b) and 2(d)). The former showed leptomeningeal involvement, and the latter was restricted to the subcutis. 

On immunohistochemistry, the first lesion was positive for GFAP (Figure 2(c)), the second negative (data not shown), but positive for S100 (Figure 2(d)). Approximately 10%–15% of the nuclei of the first lesion were positive for the proliferation marker Ki67 (MiB1) (Figure 2(e)) and 1% of the nuclei of the neurofibroma (data not shown). 

Approximately 30% of the cells in the first lesion showed immunopositivity for p53 (Figure 2(f)).

Upon recovery from surgery, the patient underwent both radiotherapy and chemotherapy. Six months later, MR-imaging showed progression of the tumor. After another six months metastases were present in the right frontal lobe and spine. The patient died shortly thereafter.

## 3. Discussion

NF1 patients have an increased risk of developing central nervous system malignancies [5]. Besides optic pathway gliomas that arise in 15% of NF1 patients before the age of 7 [10], these patients are at increased risk for developing other astrocytomas. Even though the mean age at diagnosis has been reported to be 4.5 years, the risk of developing astrocytomas persists into adulthood [11]. 

This risk is accompanied by a 5-fold increased incidence of high-grade astrocytoma [9]. Several predisposing factors have been suggested: optic pathway glioma, hyperintense lesions on a T2-weighed MRI, and hereditary factors [12, 13]. However, it is still impossible to predict the development and localization of brain tumors in NF1-patients.

Mutations at the NF1 gene, which is a tumor suppressor gene located on chromosome 17q11.2, can partly explain the clinical susceptibility for malignancies. The NF1 gene product neurofibromin functions in part as a negative regulator of the p21 Ras proto-oncogene by accelerating the conversion of active guanosine triphosphate- (GTP-)bound Ras to its inactive guanosine diphosphate- (GDP-)bound form. Active Ras, as a result of reduced or absent neurofibromin expression, leads to increased cell growth and facilitates tumor formation [14]. This growth regulatory function of neurofibromin cannot fully explain the discrepancy between some tumors in NF1 patients that stay quiescent for many years and others that rapidly change toward malignancy. It has been suggested that mutations in other genes may cooperate with NF1 mutations in the cancerogenesis observed in these patients. For most tumors found in NF1 patients, the genetic events contributing to their development remain to be elucidated. Molecular analysis of GBMs arising in NF1 patients showed the presence of genetic alterations such as p16INK4A/ARF deletions and p53 mutations, which are often also present in sporadic GBMs [8]. These alterations are believed to cooperate with NF1 loss in the development of malignant astrocytomas. 

Several groups have confirmed the cooperation of mutations in NF1 and p53 in the development of astrocytomas in transgenic mice [15, 16]. Zhu et al. reported that in their mouse model with simultaneous loss of p53 and NF1, most tumors arose in the vicinity of the subventricular zone (SVZ), where the majority of neural precursor cells reside [16]. This might indicate two things: first, cells in this region of the brain are more susceptible to p53/NF1-mediated tumor formation. Interestingly, it has been suggested that even though cells in different regions of the brain might seem morphologically identical, they are in fact unique. For example, neural precursor cells isolated from different regions of the human embryonic brain exhibit distinct growth properties [17]. Moreover, neocortical and cerebellar astrocytes display different biological and electrophysiological properties [18–23]. Finally ependymomas and pilocytic astrocytomas arising in supratentorial versus infratentorial regions of the brain display considerably different gene expression profiles [24, 25]. Second, the micromilieu of the SVZ is more advantageous to the growth of brain tumor precursor cells. Most high-grade astrocytomas in NF1 patients are found in supratentorial regions of the brain. Here, we present an NF-1 patient who developed a GBM in the posterior fossa.

As in the NF1/p53 transgenic mouse model, the GBM tumor in this NF1 patient also showed loss of p53. The cerebellar location of this tumor seems to be associated with a pre-existing lesion, which may have created an environment conducive to mutagenesis and/or supportive for tumor initiating cells. 

The characteristics of this single case suggest that brain-lesions in NF1-patients should be closely monitored for potential development of high-grade astrocytomas. A similar suggestion was made earlier by Carella et al. [26]. However, larger studies will be necessary to establish a firm correlation between lesions and development of high-grade astrocytomas in NF1 patients.
